# Predicting Endocrine Disruption Using Conformal Prediction – A Prioritization Strategy to Identify Hazardous Chemicals with Confidence

**DOI:** 10.1021/acs.chemrestox.2c00267

**Published:** 2022-12-19

**Authors:** Maria Sapounidou, Ulf Norinder, Patrik L. Andersson

**Affiliations:** 1 Chemistry Department, Umeå University, 901 87 Umeå, Sweden; 2 Department of Computer and Systems Sciences, Stockholm University, Box 7003, 164 07 Kista, Sweden; 3 MTM Research Centre, School of Science and Technology, Örebro University, 701 82 Örebro, Sweden; 4 Department of Pharmaceutical Biosciences, Uppsala University, Box 591, 75 124 Uppsala, Sweden

## Abstract

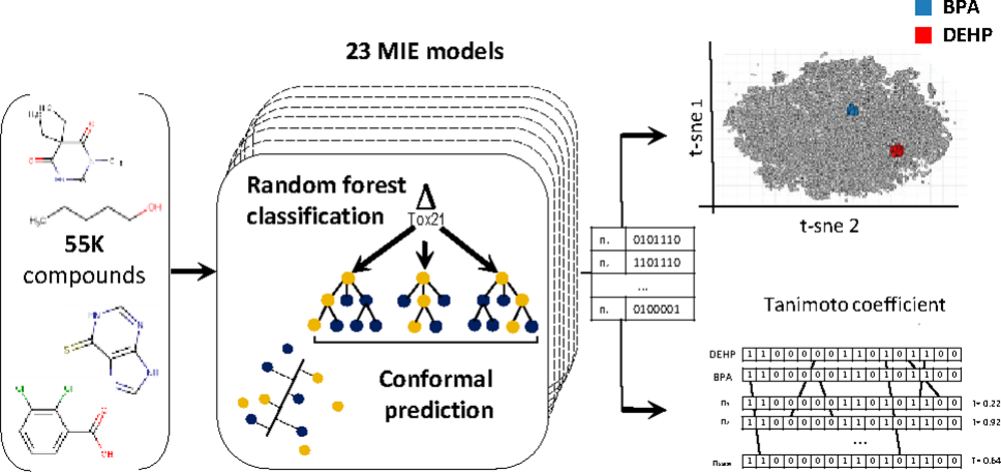

Receptor-mediated molecular initiating events (MIEs) and their relevance in endocrine activity (EA) have been highlighted in literature. More than 15 receptors have been associated with neurodevelopmental adversity and metabolic disruption. MIEs describe chemical interactions with defined biological outcomes, a relationship that could be described with quantitative structure–activity relationship (QSAR) models. QSAR uncertainty can be assessed using the conformal prediction (CP) framework, which provides similarity (i.e., nonconformity) scores relative to the defined classes per prediction. CP calibration can indirectly mitigate data imbalance during model development, and the nonconformity scores serve as intrinsic measures of chemical applicability domain assessment during screening. The focus of this work was to propose an *in silico* predictive strategy for EA. First, 23 QSAR models for MIEs associated with EA were developed using high-throughput data for 14 receptors. To handle the data imbalance, five protocols were compared, and CP provided the most balanced class definition. Second, the developed QSAR models were applied to a large data set (∼55,000 chemicals), comprising chemicals representative of potential risk for human exposure. Using CP, it was possible to assess the uncertainty of the screening results and identify model strengths and out of domain chemicals. Last, two clustering methods, t-distributed stochastic neighbor embedding and Tanimoto similarity, were used to identify compounds with potential EA using known endocrine disruptors as reference. The cluster overlap between methods produced 23 chemicals with suspected or demonstrated EA potential. The presented models could be utilized for first-tier screening and identification of compounds with potential biological activity across the studied MIEs.

## 1 Introduction

Chemicals with endocrine disruptive (ED) properties have been associated with a multitude of effects, such as neurodevelopmental interference in early life (e.g., birth weight, behavioral development^1^) or contributing to metabolic disorder development.^2^ Endocrine disrupting chemicals (EDCs) have been shown to interfere with hormonally regulated processes directly through binding to target receptors or indirectly by interacting with components of the endocrine pathways.^3^ The exact mechanistic pathways leading to ED events are yet to be elucidated; however, an OECD conceptual framework for testing and assessment of endocrine disrupters has been proposed.^4^ Within this framework, molecular initiating events (MIEs) related to estrogen receptor (ER) isoforms, androgen receptor (AR), thyroperoxidase inhibition/transthyretin binding, and retinoid X receptor (RXR) are proposed as *in vitro* endpoints to be assessed. The inclusion of *in silico*-derived data as Level 1 information is encouraged,^4^ and principles to ensure good practice of quantitative structure–activity relationship (QSAR) model development for regulatory purposes have been proposed.^5^ In the guidance,^5^ it is proposed that a QSAR for regulatory purposes should be associated with: (1) defined endpoint, (2) be based on unambiguous algorithm, (3) have a defined domain of applicability, (4) report goodness-of-fit, robustness, and predictive capacity, and (5) if possible have a mechanistic interpretation that is described with proposed QSAR.

Currently, QSAR models on endocrine activity (EA) and MIEs involving ER (e.g., CERAPP),^10^ AR (e.g., CoMPARA),^11^ pregnane X receptor (PXR),^12−15^ and thyroid receptors (TR)^16,17^ are readily available. Open-access platforms such as QSAR toolbox (e.g., ER profiler)^18^ and VEGA-QSAR^19^ (e.g., ER, TR, RXR) or standalone models from peer-reviewed literature are easily deployable with adequate supporting documentation for all implemented models. The implemented models have well-defined endpoints and are based on unambiguous algorithms such as multiple linear regression, partial least-squares regression, and k-nearest neighbors. Therefore, to meet the proposed principles on considering *in silico*-derived data for regulatory assessment of EDCs, employed models should be assessed in terms of (a) domain of applicability (principle 3) and (b) measures of goodness-of-fit, robustness, and predictive capacity prior to deployment (principle 4).

The applicability domain of a model is dictated by the data set it is based on; the more diverse the data set, the wider the applicability domain of a model. There is restricted availability of large and chemically diverse data sets on endpoints related to ED events. For this reason, the majority of current *in silico* modeling efforts on EA prediction employ data from the initiatives Tox21 and ToxCast. Within these initiatives, *in vitro* quantitative high-throughput screening (qHTS) data have been generated for approximately 10,000 compounds.^6^ Previously, model development limitations have been reported due to data skewness, discrepancies of reported bioactivity among replicates, and overlooked cytotoxicity results when curating receptor activity assay data.^7^ In turn, successful curating strategies have been suggested (e.g., Judson et al.^8^) and applied (e.g., Gadaleta et al.^9^).

Principle 3 should be also evaluated in conjuction with principle 4 when QSARs are considered for regulatory assessment of EDCs. Principle 4 refers to model performance during development and validation. To ensure predictions of high confidence, it is necessary to evaluate whether a chemical of unknown activity falls within a model’s applicability domain. To facilitate this evaluation and address prediction uncertainty, QSAR models should report measures of goodness-of-fit per prediction. VEGA-QSAR prediction reports disclose whether chemicals of interest are within the training set and indices to evaluate applicability.^19^ However, several QSAR toolbox profilers do not provide explicit quantifiable confidence measures per prediction or clear definition of the chemical applicability domain.

Conformal prediction (CP) is a mathematical framework that provides measures of uncertainty for predictions derived from an *in silico* model.^20^ Additionally, it has been demonstrated that CP implementation could improve imbalance of class definition caused by data set skewness.^21,22^ It was hypothesized that implementation of CP could improve the aforementioned limitations on EA prediction. The objectives of this work were (a) to develop *in silico* models for endocrine activity intended for first-tier screening and and (b) to propose a strategy to identify chemicals with EA potential using clustering methodologies. To meet objective (a), 14 receptors were identified to be involved in MIEs associated with neurodevelopmental and metabolic disrupting adverse effects by Lupu et al.^23^ and Legler et al.^24^ For these receptors, data sets from Tox21 qHTS bioassays were retrieved and curated. Twenty-three *in silico* models were developed using the Random Forest Classification algorithm. Five protocols that handle imbalanced data sets, including CP, were applied and compared to assess the effectiveness of CP on class definition. Next, all developed QSAR models were applied to a large data set relevant to human exposure (∼55,000 chemicals), and predictions with defined uncertainty measures were derived and discussed. To meet objective (b), it was hypothesized that application of clustering methodologies and visualization using bioactivity as criterion of similarity could produce chemicals of potential EA activity. Predicted bioactivity profiles of the data set from objective (a) were compared with bioactivity profiles of two known EDCs using two clustering methods, t-distributed stochastic neighbor embedding (t-SNE) and Tanimoto similarity.

## 2 Methodology

### 2.1 Data Sets

Legler et al.^24^ and Lupu et al.^23^ discussed the strong links of MIEs involving aryl hydrocarbon receptor (AhR), AR, constitutive androstane receptor (CAR), estrogen receptor alpha (ER-α), farsenoid X receptor (FXR), glucocorticoid receptor (GR), peroxisome proliferator-activated receptor gamma (PPAR-γ) and delta (PPAR-δ), progesterone receptor (PR), PXR, retinoic acid receptor (RAR), RXR, thyroid hormone receptor (TR), and vitamin D3 receptor (VDR3) with ED adverse effects. Data sets from bioassays that identify agonistic and antagonistic activity of small molecules with proposed receptors were selected (Table S1). As part of the U.S. Tox21 Program, summaries of bioassay records were released, which combined results from receptor activity assays and cell viability counter screens. For the presented work, the PUBCHEM_ACTIVITY label was used to indicate an active or inactive compound. For each unique PubChem CID with multiple results (Active, Inactive, or Inconclusive), only those with a majority activity (Active, Inactive) decision ≥2/3 were included. A cutoff of 0.3% active/inactive ratio was set as the sole exclusion criterion. In total, 23 assays were included (Table 1) and only 2 were excluded (i.e., TR agonism and VDR3 agonism) from further analysis.

**Table 1:** Active/Inactive Ratios for Receptor Binding Bioassays Within the U.S. Tox21 Initiative Considered for Model Development^a^

Target Receptor	Molecular Initiating Event	Data set	Active/Data set (%)
AhR	activation	6671	10.94
AR	agonism	7130	3.00
	antagonism	6286	6.28
CAR	agonism	6629	11.80
	antagonism	5059	2.49
ER-α	agonism	7242	4.42
	antagonism	6287	4.23
FXR	agonism	6812	1.16
	antagonism	6114	2.60
GR	agonism	7116	2.04
	antagonism	6167	4.70
PPAR-δ	agonism	6455	1.02
	antagonism	6204	0.77
PPAR-γ	agonism	6795	2.56
	antagonism	5915	4.90
PR	agonism	7347	1.46
	antagonism	6201	12.01
PXR	agonism	6144	24.25
RAR	agonism	5916	5.04
	antagonism	4919	9.53
RXR	agonism	5566	2.61
TR-β	antagonism	5554	4.65
VDR3	antagonism	6007	0.78

a AhR: aryl hydrocarbon receptor; AR: androgen receptor; CAR: constitutive androstane receptor; ER-α: estrogen receptor alpha; FXR: farsenoid X receptor; GR: glucocorticoid receptor; PPAR-γ: peroxisome proliferator-activated receptor gamma; PPAR-δ: peroxisome proliferator-activated receptor delta, PR: progesterone receptor, PXR: pregnane X receptor, RAR: retinoic acid receptor, RXR: retinoic acid receptor; TR-β: thyroid hormone receptor; and VDR3: vitamin D3 receptor.

Chemicals associated with human exposure (referred as human exposure risk, HER) had been compiled by Mansouri et al.^11^ HER was selected due to its size (*n* = 55,337 chemicals), and its inclusion of metabolic structures (*n* = 6592) with predicted estrogenic activity, whose parent compounds have predicted nonestrogenic activity.^11^ HER comprise chemicals from sources such as the European inventory of existing commercial chemical substances.^25^ HER curation has been already described, which included QSAR-ready SMILES standardization.^10,11^ To further ensure data quality, PubChemIDs were randomly selected using the KNIME node Random Number Assigner to reach 50 and 100 chemicals from the training and HER data set, respectively. All 150 chemicals were manually checked and matched successfully with the reported QSAR-ready SMILES.

### 2.2 Model Development and Performance Assessment

#### 2.2.1 Model Development Protocol

Using RDKit descriptors, models were developed following a stratified 5-fold cross validation protocol, and the Random Forest classification (RFC) algorithm. RFC was performed using Gini Index and 200 trees.

#### 2.2.2 Handling Data Imbalance

To account for data imbalance (Table 1), five protocols were tested and implemented in the RFC protocol: conformal prediction (CP) equal size sampling (under-sampling), over-sampling by duplication, by synthetic minority over-sampling technique, and by random over-sampling examples, and (details on Table 2). A naïve protocol was also performed as control.

**Table 2:** Description of Tested *In Silico* Protocols

Protocol	Implementation and settings	Method description
Naïve		No class imbalance class handling
Equal size sampling (under-sampling)	KNIME node equal size sampling. Set to exact match of classes	Node removes random rows from majority class to match the size of the minority class.
Duplication – over-sampling	KNIME	Increase of minority class by duplication of real objects.
Synthetic minority over-sampling technique (SMOTE)	KNIME node SMOTE. Set to identify 5 nearest neighbors	From the data set, the algorithm pairs real object with nearest neighbor from same class, selects a random point between neighbors, and populates synthetic rows with attributes based on this randomly selected point^26^
Random over-sampling examples (ROSE)	R script package. Default parameters	The algorithm produces synthetic rows to enlarge both the minority and majority class size, with artificial data derived by a conditional kernel density estimate of the two classes^27^
Conformal prediction (CP)	Python (data availability)	Mathematical framework for machine learning models, that provides measures of uncertainty (see more details in Section 2.2.3)

#### 2.2.3 Conformal Prediction

CP is a mathematical framework to quantify confidence of *de novo* predictions. For a thorough and detailed description of CP, see previous studies using the method,^28−30^ and for an in-depth analysis of the mathematical and statistical theorems behind CP see the work by Vonk et al.^31^

**Figure 1:**
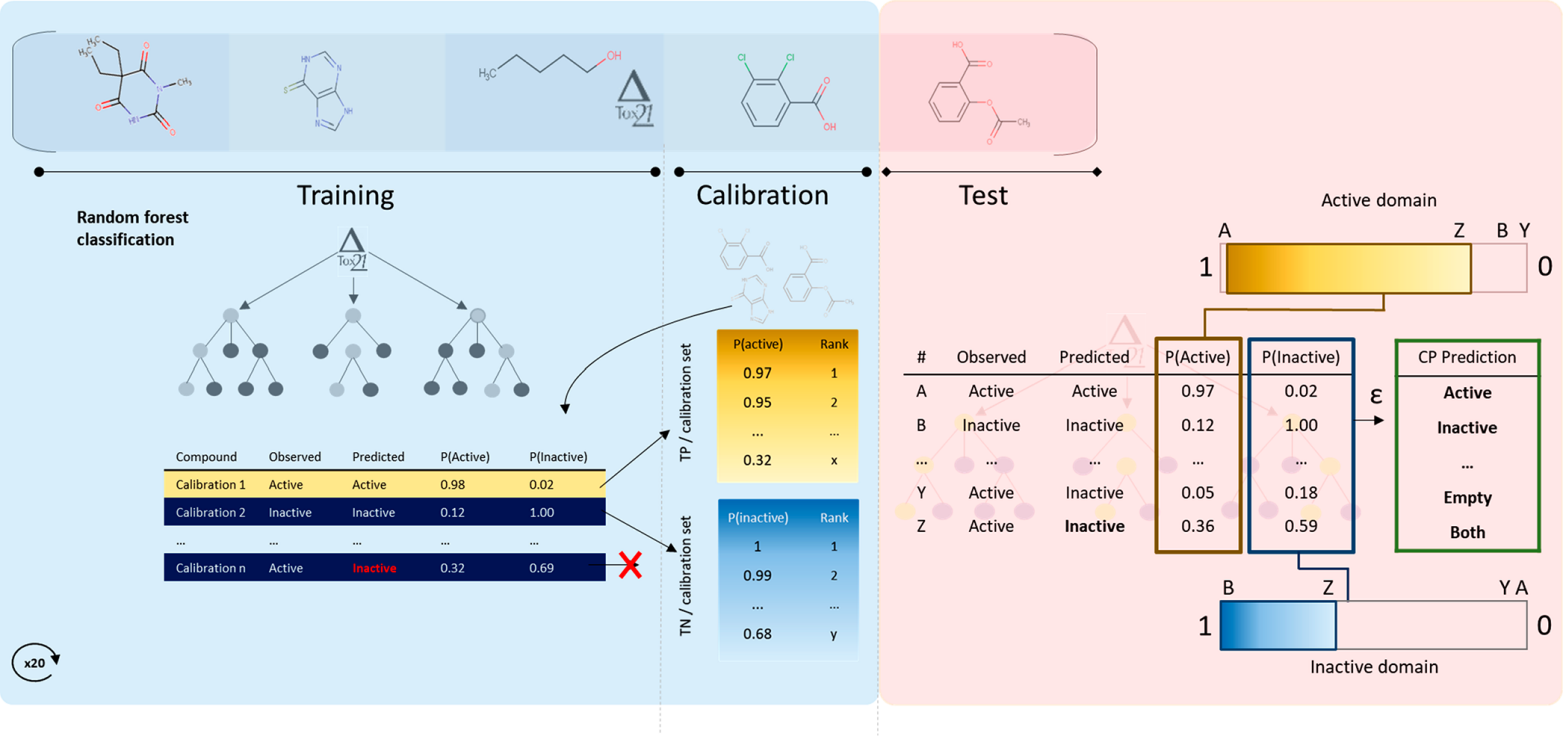
Conceptual representation of RFC and CP model development and evaluation. Per fold, 20% was reserved for test set, 60% for training, and 20% for calibration. In the model development stage (blue background), the trained RFC-model is applied to the calibration set. For the successfully predicted compounds of the calibration set, RFC derived *p*-values are collected and ranked in the respective classes (i.e., active domain calibration table and inactive domain calibration table). During the validation step (red background), the trained RFC-model is applied to the test set, and RFC derived *p*-values are compared with the active and inactive domain independently. Based on the relative ranking in the respective domains, nonconformity scores (i.e., *p*1 and *p*0) are calculated. Finally, class assignment is based on the defined acceptable error of significance (ε).

The CP framework introduces two elements in a model development protocol: calibration and definition of local levels of confidence (Figure 1). Using CP, the trained RFC model is applied to the calibration set, for which activity of the compounds is known. In the calibration tables, compounds are ranked based on, in this case, decreasing probabilities by the model. The calibration tables serve as the ground for setting local levels of confidence as well as for determining the respective *p*-values (one *p*-value for each class). In a binary classification model, *p*-values are assigned for each class classes (i.e., *p*-active and *p*-inactive) that quantify similarity (i.e., conformity) within the respective class, e.g., for an active compound, it would be expected *p*-active≥ significance level and *p*-inactive < significance level. The class balancing effect in CP is achieved by comparing each class independently as two separate distributions. As in any other modeling effort, calibration tables reflect the quality of the training set on class definition. When deploying the model, predicted probabilities attributed to new chemicals are ranked within each calibration table. The rank of the tested compounds within each of the calibration tables can be translated into confidence, i.e., the higher the *p*-value of a new compound within a class, the higher the similarity assumed with the corresponding class, the higher the confidence on the assignment. For class assignment, both *p*-active and *p*-inactive values are used. For compound X with *p*-active value 0.95 and *p*-inactive 0.01, the class assignment would be ‘active’.

There are three potential outcomes for class assignment (Figure 1): (a) ‘active’ or ‘inactive’ (i.e., single class assignment), (b) assignment to ‘both’ classes (i.e., for the defined error rate (significance level), distinction between classes is not possible), and (c) ‘empty’ (i.e., classification not possible for the selected local confidence levels, ‘out of domain’ classification). For a more detailed description on how this calibration is performed, see Norinder et al.^28^

Definition of local levels of confidence is equally crucial during the calibration step, because it dictates the final class assignment. In CP, local levels of confidence are user-defined, and they are referred to as acceptable error of significance. In a scenario of 0% acceptable error, if class assignment was ‘both’ for all compounds, it would be considered correct. Hence, definition of the acceptable error should be informed by two CP-specific measures, efficiency and validity. Efficiency represents the ratio of single class predictions per class, and validity represents the ratio of correctly assigned compounds per class. For screening, it is optimal both measures both measures to be above 0.80 (i.e., 80% of the predictions are correctly assigned, and are assigned to a single class). Here, CP was performed in 10 iterations (Figure 1), and the calibration set was randomly sampled for each iteration.

### 2.3 Model Statistical Analysis

Statistical parameters for binary classification were calculated for all developed models (e.g., accuracy, sensitivity, specificity, F-measure, Matthew’s correlation coefficient (MCC), positive (PPV) and negative predictive value (NPV), area under the ROC curve (AUC)) with the Binary Scorer KNIME node, which accounted for out of domain and unequivocal class assignment. For models developed with CP, model performance was assessed on five levels of significance (i.e., 0.1, 0.15, 0.2, 0.25, 0.3), and measures of validity and efficiency per class per model were calculated (Table S3). It should be stressed that model assessment parameters for CP were calculated for single label predictions, i.e., active or inactive, only.

### 2.4 HER Screening and Chemical Similarity Strategies

HER screening following naïve and under-sampling protocol were performed using KNIME, and following CP using Python. Analysis of screening and clustering results, including identification of commonly occurring fragments (node MoSS), were conducted using KNIME.^32^ Nonconformity *p*-values (i.e., *p*1, *p*0) of HER data set were derived and reported in Table S4.

**Figure 2:**
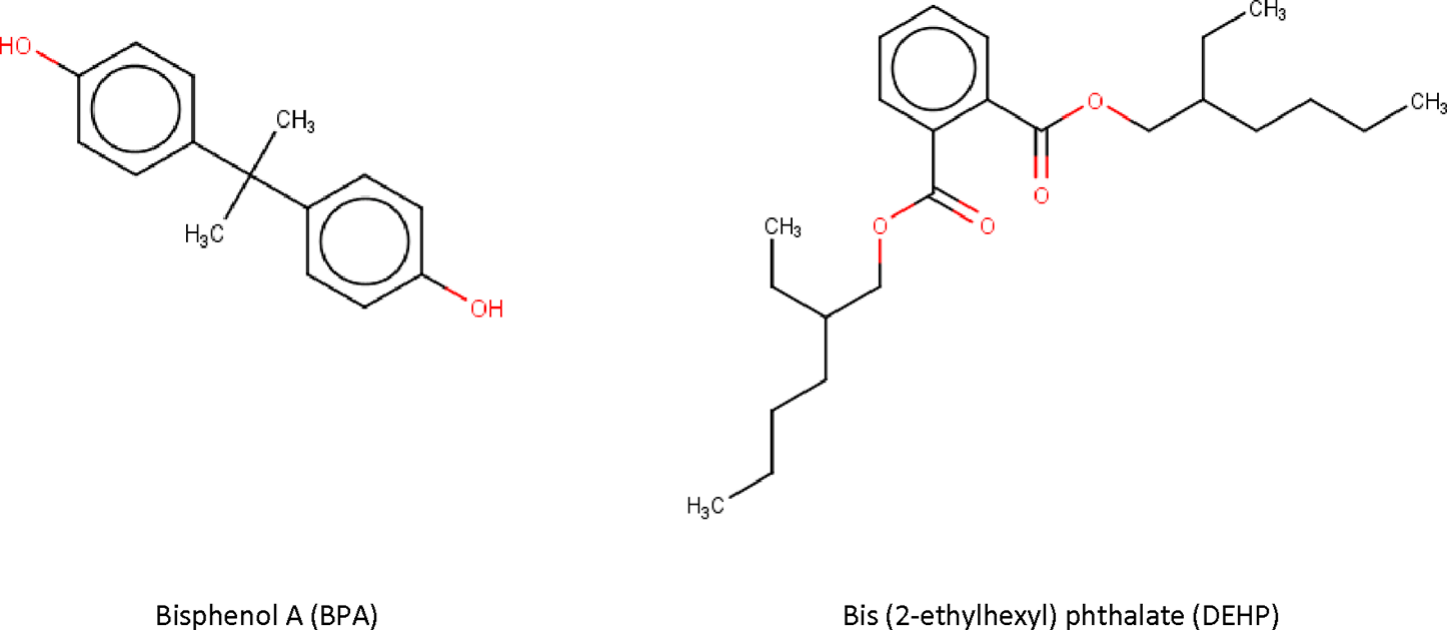
Chemical structures of BPA and DEHP.

To assess whether *in silico* methodologies can support chemical prioritization, two similarity methods were applied, t-SNE and Tanimoto similarity. Comparison was based on predicted nonconformity *p*-values across all developed models using as reference, known endocrine disruptors. Bisphenol A (BPA)^33^ and bis(2-ethylhexyl) phthalate (DEHP)^34^ were selected as reference compounds. These compounds were selected, because they are industrial organic compounds; they have documented ED activity relevant to both human and environmental health; and they are among the first compounds within EU to be classified as chemical of concern due to their endocrine disrupting effects^35^ (Figure 2).

t-SNE is an unsupervised nonlinear probabilistic exploratory and visualization algorithm, which embeds high-dimensional data for visualization in a low-dimensional space.^36^ In brief, the principle behind t-SNE is calculation and comparison of probabilities of proximity in higher- and lower-dimensional space. This comparison is the premise for visualization, where the differences are attempted to be minimized in a lower-dimensional space, using local minima by applying a gradient descent. A detailed explanation of the method is presented by van det Maaten and Hinton.^37^ For the purposes of this work, t-SNE was performed, following the protocol openTSNE (version 0.3.11)^36^ in Python environment (version 3.7) using conformal *p*-values as input features for the studied data set.

Tanimoto coefficients are scores that represent similarity between sets of elements such as fingerprints in binary. For this comparison, fingerprints in binary (1/0) were constructed based on conformal *p*-values (*p*1, *p*0) across 23 endpoints. If *p*1 > *p*0, a value 1 was set, and if *p*1 < *p*0, a value 0 was set; values 1 and 0 express the largest *p*-value and not class assignment. This generated a 23-bit long (1/0) fingerprint (e.g., 00110111001100010110111) for each compound. Two Tanimoto scores were calculated for all HER compounds when compared with BPA and DEHP, respectively.

## 3 Results and Discussion

### 3.1 *In Silico* EA-Specific Screening Battery

In the current study, 23 classification models were developed based on curated Tox21 data sets of EA-relevant endpoints. Training skewness was a common feature for all data sets, where the active domain represented 0.78 to 24.25% (median 4.23) of the data set (Table 1). Proposed strategies that handle data imbalance focus on minority class increase (e.g., *n*-fold over-sampling, active learning^38^), majority class reduction (e.g., under-sampling), synthesis of artificial data (e.g., SMOTE,^26^ ROSE^27^), and/or model structure tailoring (e.g., subsampling and ensemble QSARs^39^). CP has been also shown to handle data skewness in a number of examples (e.g. refs (29 and 40)).

In agreement with previous studies,^29,40^ CP provided the most balanced performing models compared to other protocols without compromising model performance (Table 3, for more details see Table S2). It is acknowledged that other protocols outperform CP in specific aspects of model performance with respect to one class (e.g., higher accuracy in individual models when following an over-sampling protocol; higher MCC score when compared to naïve), but not for both. Performance parameters, such as balanced accuracy, balanced PPV, and balanced NPV were on average above 0.8 across all 23 CP models, suggesting a balanced definition for both classes (see Tables 3 and S2).

**Table 3:** Overview of Average Performance Parameters on Test Set for Developed Models Using Random Forest Classification, Following Different Protocols for Handling Data Imbalance

Performance parameters	Naive	Under-sampling	Over-sampling	ROSE	SMOTE	CP^a^	
Balanced Accuracy	0.61	0.78	0.65	0.55	0.66	0.71^b^	0.82
Accuracy	0.96	0.78	0.96	0.19	0.96	0.69^b^	0.81
Sensitivity	0.24	0.79	0.31	0.94	0.34	0.73^b^	0.83
Specificity	0.99	0.77	0.99	0.16	0.98	0.69^b^	0.81
Balanced PPV	0.98	0.78	0.97	0.54	0.97	0.81	
Balanced NPV	0.57	0.79	0.60	0.79	0.61	0.83	
MCC	0.38	0.28	0.41	0.07	0.42	0.34	
AUC	0.85	0.53	0.18	0.71	0.19	0.88	
Coverage	1	1	1	1	1	1	0.89

a Acceptable error level of significance is 0.2 for CP models.

b Including chemicals that are ‘empty’ or ‘both’.

Generally, data imbalance is reflected on the skewness of predictability between classes regardless of tested protocol (i.e., mean MCC = 0.32), with high levels of specificity (e.g., over-sampling: 0.99), and low levels of sensitivity (e.g., naïve: 0.24) (Table 3). For CP models, training skewness influenced performance, with expected false positive and false negative rates to be ∼20% (i.e., balanced PPV and NPV ∼0.8). For CP models, the optimum acceptable error signified that correct classification was derived for more than 80% (validity:0.8) of the test set, and single class assignment was derived for more than 80% of the test set (efficiency:0.8).

When comparing model performance, it is important to reiterate a key distinction of CP class assignment over other protocols. CP class assignment is based on user-defined levels of acceptable error (significance level), which defines local levels of confidence and influence model coverage (Table 3). This distinction hinders straightforward comparison of model performance with the other tested protocols, where a single label classification is the default outcome of the majority vote. CP performance parameters in Table 3 are reported in reference to the whole data set (coverage 1) and adjusted to CP coverage as well (coverage 0.89). CP class assignment provides transparently model limitations, which are not readily available for most other protocols and which demand expertise to attain. Derived average AUC was 0.88, whereas among other tested protocols, the mean AUC was 0.49 (0.19–0.85). This example demonstrates the essentiality of transparent intrinsic measures to assess uncertainty in model evaluation and prediction.

Among the 23 models developed with CP, 15 demonstrated high predictability (efficiency and balanced accuracy for both classes >0.8) and 8 moderate (efficiency and/or balanced accuracy between 0.6 and 0.8) (Table 4). The best performing models were for the MIEs AhR activation, CAR agonism, PR agonism, PR antagonism, and PXR agonism (Tables 4 and S3).

**Table 4:** Overview of Performance on Test Set for Developed Models Using Random Forest Classification and Conformal Prediction^a^

		Conformal Prediction				
Target	Endpoint	Balanced Accuracy	Efficiency	MCC	AUC	Coverage
AhR	activation	0.87	0.92	0.56	0.93	0.92
AR	agonism	0.85	0.94	0.34	0.92	0.94
	antagonism	0.85	0.99	0.39	0.97	0.99
CAR	agonism	0.86	0.94	0.55	0.96	0.94
	antagonism	0.79	0.91	0.22	0.89	0.91
ER-α	agonism	0.79	0.92	0.27	0.92	0.92
	antagonism	0.82	0.99	0.30	0.97	0.99
FXR	agonism	0.85	0.77	0.19	0.82	0.77
	antagonism	0.83	0.98	0.26	0.95	0.98
GR	agonism	0.80	0.96	0.21	0.91	0.96
	antagonism	0.85	0.96	0.36	0.95	0.96
PPAR-δ	agonism	0.81	0.80	0.17	0.65	0.80
	antagonism	0.63	0.70	0.06	0.80	0.70
PPAR-γ	agonism	0.81	0.87	0.23	0.85	0.87
	antagonism	0.79	0.95	0.30	0.93	0.95
PR	agonism	0.93	0.90	0.69	0.87	0.90
	antagonism	0.88	0.95	0.62	0.91	0.95
PXR	agonism	0.88	0.95	0.70	0.93	0.95
RAR	agonism	0.81	0.97	0.35	0.95	0.97
	antagonism	0.80	0.94	0.40	0.95	0.94
RXR	agonism	0.74	0.58	0.17	0.60	0.58
TR-β	antagonism	0.78	0.97	0.28	0.96	0.97
Vit D3	antagonism	0.86	0.58	0.19	0.78	0.58

a 0.2 acceptable error level of significance.

### 3.2 Deployment of an *In Silico* EA-Specific Screening Battery

**Figure 3:**
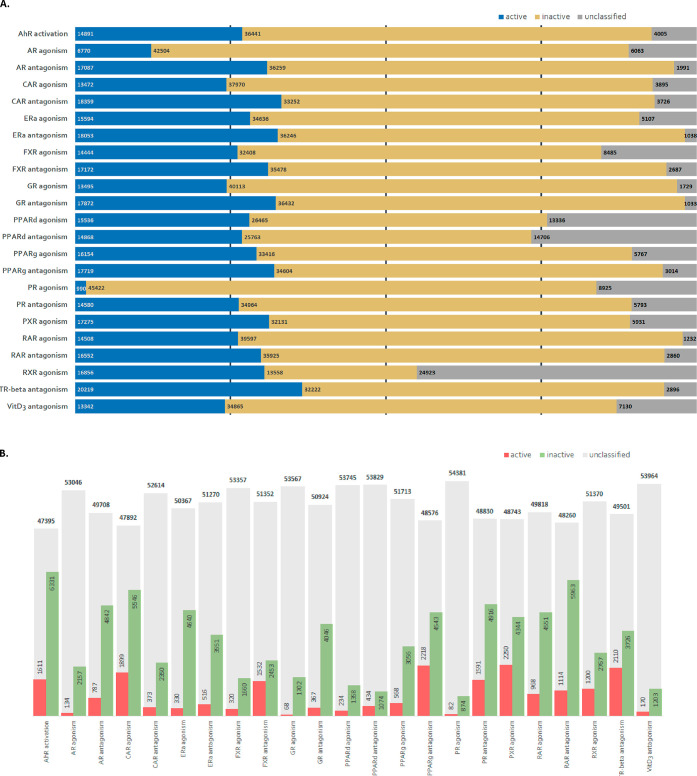
HER data set (*n* = 55337) screening results using 23 *in silico* CP-models associated with ED-linked MIEs. With CP implementation, user-defined threshold dictates class assignment, indicative of the acceptable error of significance per prediction. For threshold (A) 0.2 and (B) 0.8, HER data sets are assigned as active, inactive, or unclassified (i.e., ‘unequivocal’ and ‘empty); note: scales are not proportional for the bars.

To evaluate in practice how CP could contribute to a first-tier screening scenario, all 23 models were applied to the HER data set. At first glance (Figure 3A), 27% is predicted on average as active, 62% as inactive, and 11% as unclassified (i.e., classified as ‘both’ or ‘empty’) across models.

The highest levels of predicted actives were reported from the TR-β antagonism model (∼36%) and lowest from the PR agonism model (∼2%) (Figure 3A). Differences in active class assignment (%) between training and predicted were expected (see Section 3.1 and Table S4). At most, 20% of the predicted active domain was expected to be false positives, accounting for model parameters such as balanced PPV and set acceptable error of significance. On average, prediction of active compounds in HER data set is 10-fold higher than expected, accounting for balanced PPV and expected false positive rate this difference falls to 8-fold (Tables S4 and S5). The PXR agonism and PR agonism models are expected to have the lowest number of false positives results (i.e., ∼1.2-fold difference predicted/expected actives), and the highest from the models on VDR3 antagonism and PPAR-δ antagonism (i.e., >30-fold). Reasons for these discrepancies could be attributed to training skewness, receptor promiscuity (e.g., AR;^11,41^ CAR;^36^ GR^37^), or bias due to differences between model data set and HER. However, it is assumed that model data sets and HER are sufficiently similar, and for the receptors with documented promiscuity, the fold difference between expected and predicted active is low.

Interestingly, the highest discrepancies between predicted and expected actives are derived from models with limited active domain (i.e., 47–79 compounds) (Tables 1 and S4). Even though data imbalance was addressed with CP, inherent limitations of the data set and its active domain could not. It should be also noted that model performance was not indicative of this discrepancy, since high false positive rate was expected for models with adequate performance parameters as well, e.g., FXR agonism model (Tables 4, S3, and S4). A false positive class assignment could be considered prudent and aligned with the precautionary principle; however, it misses to provide valuable insights to actively inform prioritization strategies.

CP implementation provides an additional level of information, because classification is based on quantifiable measures of similarity with the respective classes (i.e., conformal *p*-values: *p*1, *p*0). Class assignment with CP is dictated by the user-defined acceptable error of significance (ε), but conformal *p*-values are not. Therefore, it is possible to follow different chemical selection strategies and tailor class definition criteria depending on the purpose of the modeling. Below it is discussed how to derive high confidence predictions and ‘out of domain’ chemicals.

Compounds are predicted as active, when *p*1 is above assigned acceptable significant error and *p*0 below that. Consequently, it is possible to adjust the threshold and identify compounds predicted with high similarity within a class by increasing the ε if, e.g., candidates for costly experimental testing are to be identified. A stricter class assignment (i.e., ε > 0.8) shifted the distribution among classes, a significant decrease of efficiency, and increase of unclassified compounds (Figure 3B, Table S5). For conformal *p*-values > 0.8, 68–2250 (mean: 908, median: 568) compounds were predicted as active and 874–5963 (mean: 3377, median: 3551) as inactive per model (Table S4). This strategy reduced drastically the number of expected false positives and quadrupled the number of compounds suspected as actives per MIE (Table S4). The clusters of chemicals predicted as actives (Table S4) could inform prioritization testing for MIEs, when further information is needed (e.g., models with low balanced PPV or high false positive rate, Table S2), highlight chemical domains of potential concern (e.g. ref (42)), or with therapeutic applications (e.g., ref (43)).

Apart from single class assignment, CP classification outcome can be ‘both’ (i.e., both conformal values above set ε) and ‘empty’ (i.e., both conformal values below set ε), i.e., ‘out of domain’ compounds (see ‘unclassified’ in Figure 3). From the unclassified, it is possible to derive ‘out of domain’ compounds that are not sufficiently similar with the active or inactive class. Conformal *p*-values signify similarity, so it is possible to flag chemicals as ‘out of domain’, if they are not sufficiently similar with the active or inactive class, using as similarity measure their conformal *p*-values. For the presented screening results, chemicals with *p*1 and *p*0 both < 0.2 were considered ‘out of domain’ (Table S5). A great variation in ratio of out of domain chemicals was noted among the 23 models. For the more specific MIEs (e.g., PR agonism), a higher ratio of ‘out of domain’ compounds was found among unclassified (Table S5). In turn, the ‘both’ classification was the most prominent within the ‘unclassified’ for models with poorly defined active domain (e.g., FXR agonism, see above), and in these models, very few chemicals were flagged as ‘out of domain’ (Table S5).

As discussed in Section 3.1, measures of uncertainty are very useful to scrutinize first-tier screening results in a transparent way. In first-tier screening, it is preferable to have a high rate of false positives than false negatives, aligned with the precautionary principle. For further hazard assessment, additional hazard indicators, such as persistence, should be considered.

### 3.3 Chemical Similarity Strategies

**Figure 4:**
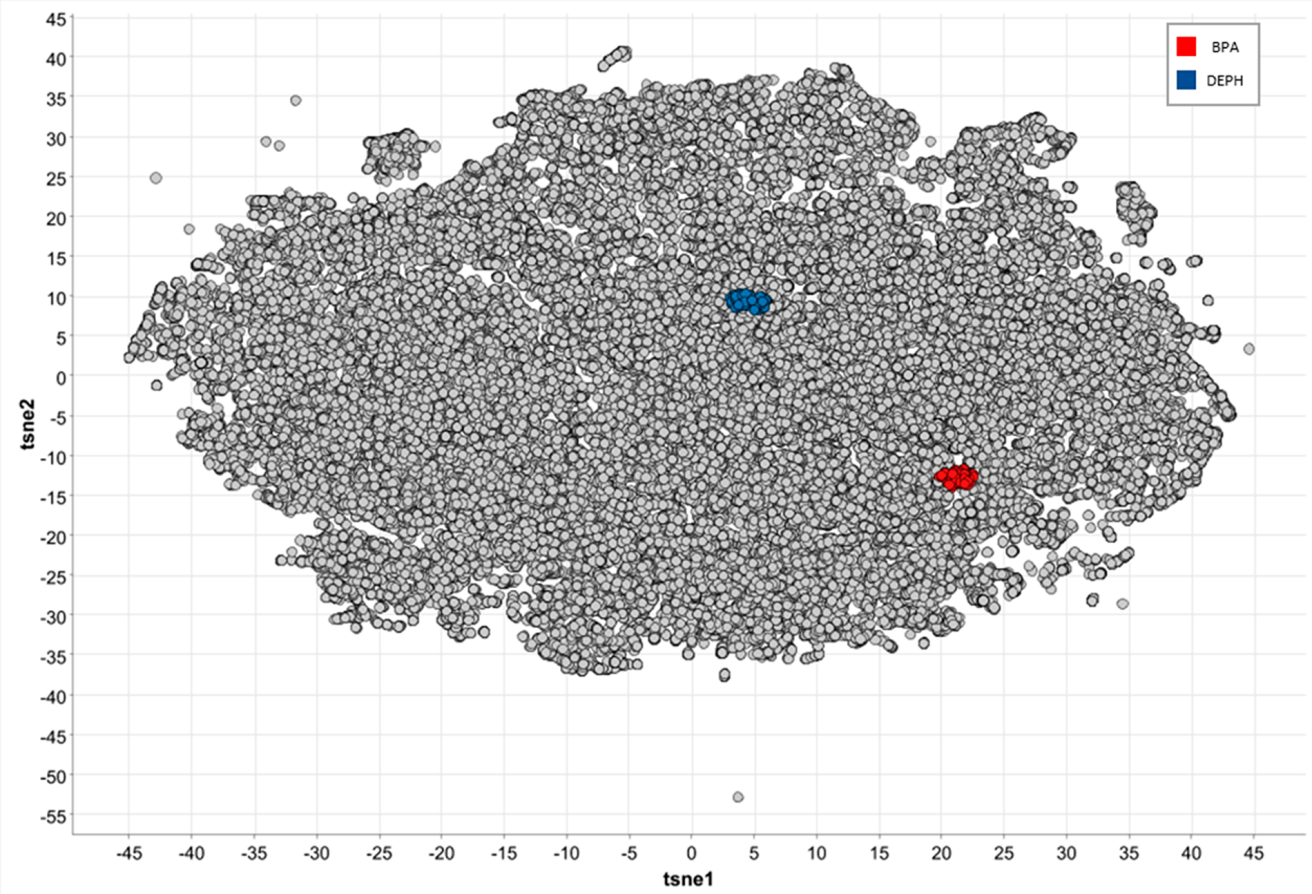
BPA (red) and DEHP (blue) t-SNE clusters as compared with t-SNE values of HER data set (gray).

**Figure 5:**
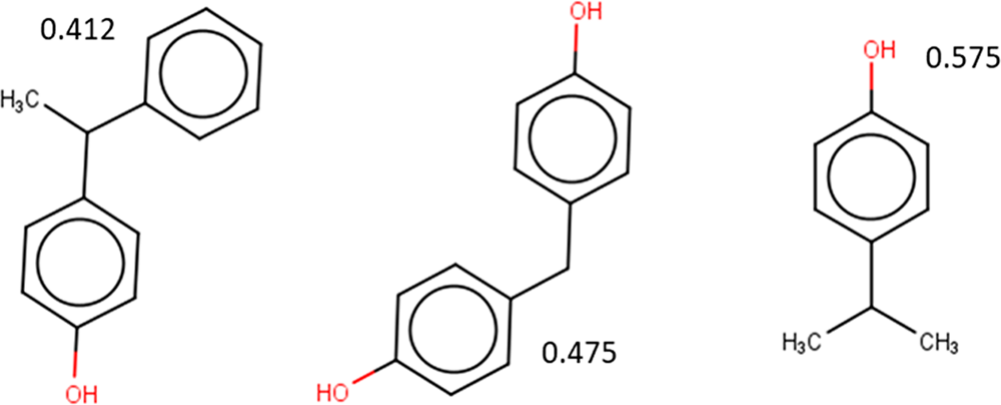
Commonly occurring structural alerts within BPA t-SNE cluster (frequency levels annotated).

**Figure 6:**
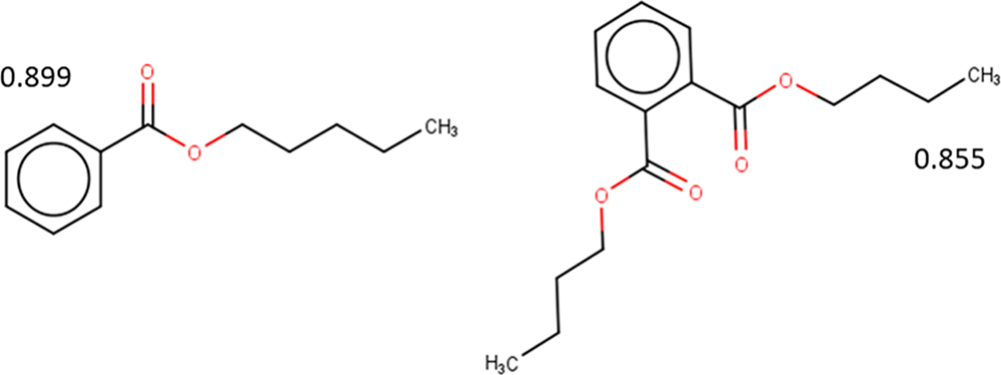
Commonly occurring structural alerts within DEHP t-SNE cluster (frequency levels annotated).

*In silico* grouping methodologies could provide a macroscopic overview and map systematically chemical and biological domains based on defined similarity criteria. Evidence suggests that EA is not driven by a single MIE and relying on SARs is not sufficient to inform prioritization. Thus, it was explored whether *in silico* similarity methodologies could support a prioritization strategy to highlight compounds of emerging concern. Predicted conformity profiles of the HER data set and reference compounds were compared using two *in silico* methods, the t-SNE method (Figures 4–6), and Tanimoto similarity (Table 5). BPA and DEHP were selected as reference compounds, because they are industrial chemicals, characterized by ECHA as EDCs with effects relevant both for human health and the environmental,^44^ and with a wealth of evidence on their ED mode of action.^33,34^

**Table 5:**
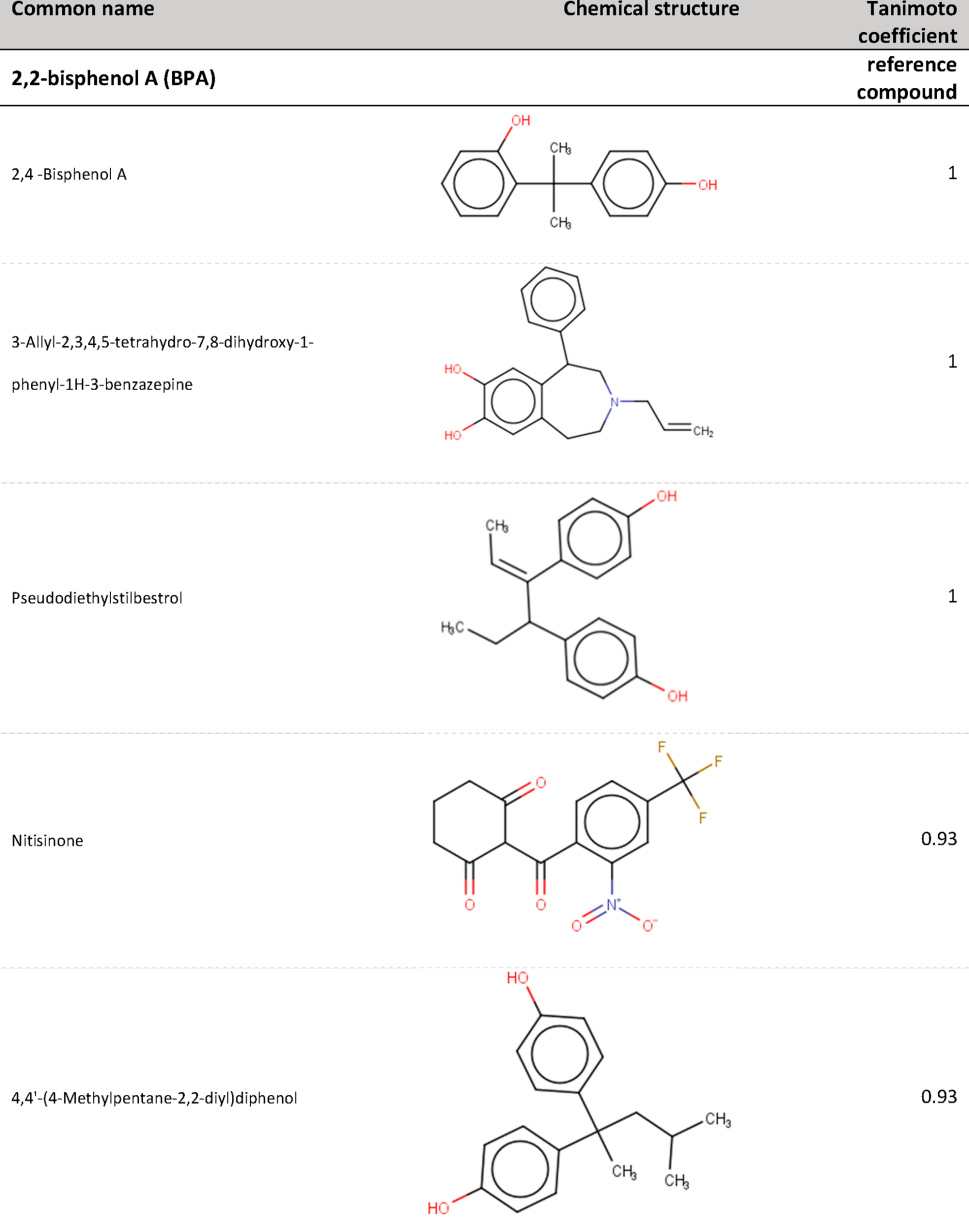

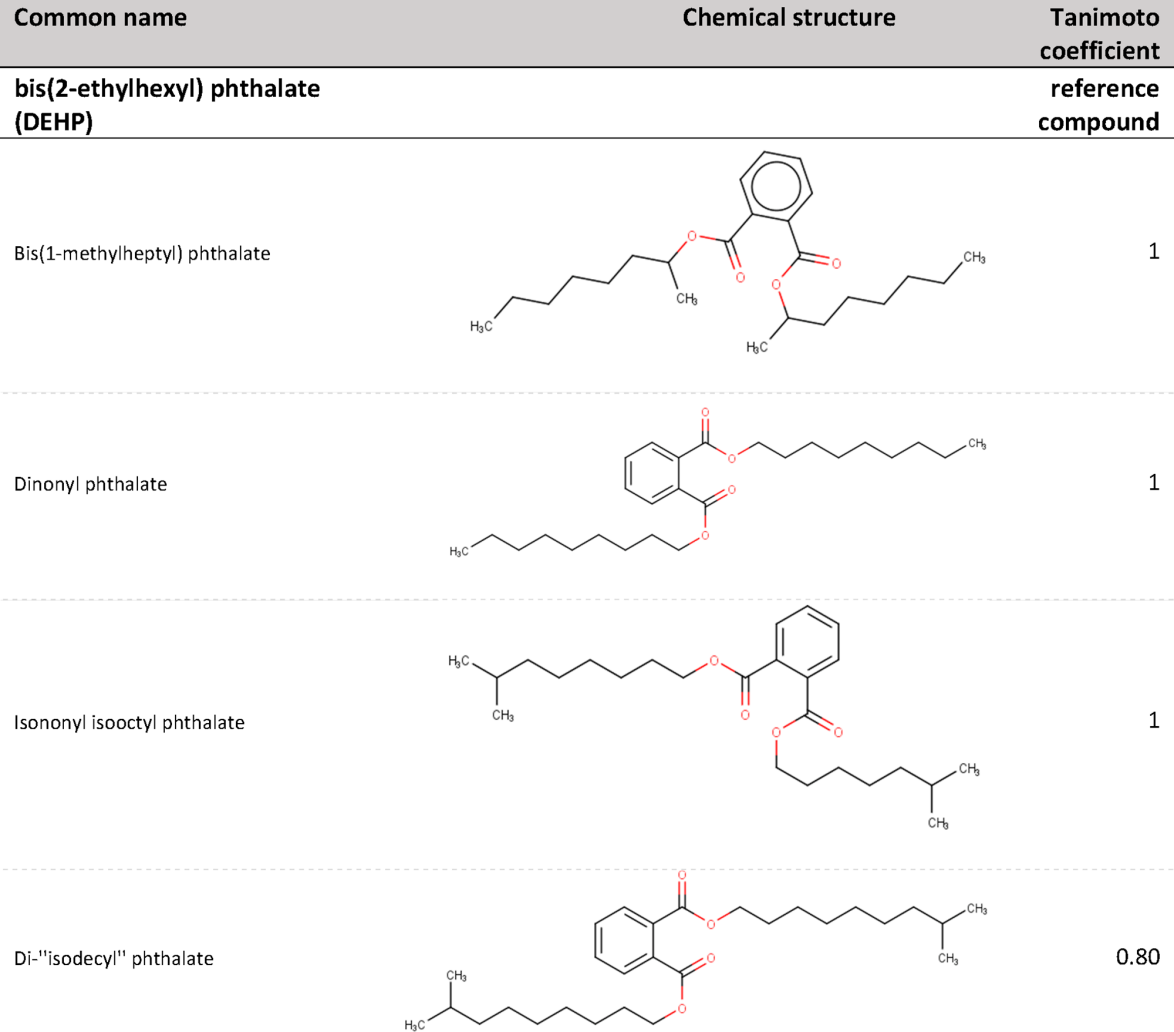
Tanimoto Coefficients for Compounds from the HER Dataset, Using BPA and DEHP as Reference Compound

t-SNE clustering revealed distinct clusters for BPA and DEHP (Figure 4, Tables S6 and S7). The t-SNE cluster for BPA comprised 80 chemicals (Table S6) with mean MW 294.5 kDa (median 289.3) and mean Slog*P* 4.47 (median 4.42). There was no striking structural homogeneity within the cluster, apart from all having 2–3 benzene rings (examples of structural alerts and their frequency levels in Figure 5). The t-SNE cluster for DEHP comprised 69 phthalates (Table S7) with mean MW 412 kDa (median 418.6) and mean Slog*P* 7.19 (median 7.29) (substructure examples with frequency levels in Figure 6).

Following the CompTox Dashboard default,^45^ 0.8 was set as cutoff to assess similarity across predicted biological activity of the previously presented models using Tanimoto coefficients. For BPA, there were 173 chemicals with Tanimoto coefficients above 0.8 among 3 had score 1 (see Tables 4 and S7). For DEHP, there were 4 chemicals with Tanimoto coefficients above 0.8, of which 3 had an identical predicted activity profile with DEHP and thus coefficient of 1 (Table 5).

Both methods indicated a number of chemicals with similar or even identical predicted bioactivity with BPA and DEHP, on MIEs related to endocrine activity. Interestingly, more than 100 of these chemicals are included in the HBM4EU screening list for chemicals of emerging concern (CECs)^46^ and/or NORMAN suspect list;^47^ evidence that supports their inclusion in EDC suspects lists.

**Figure 7:**
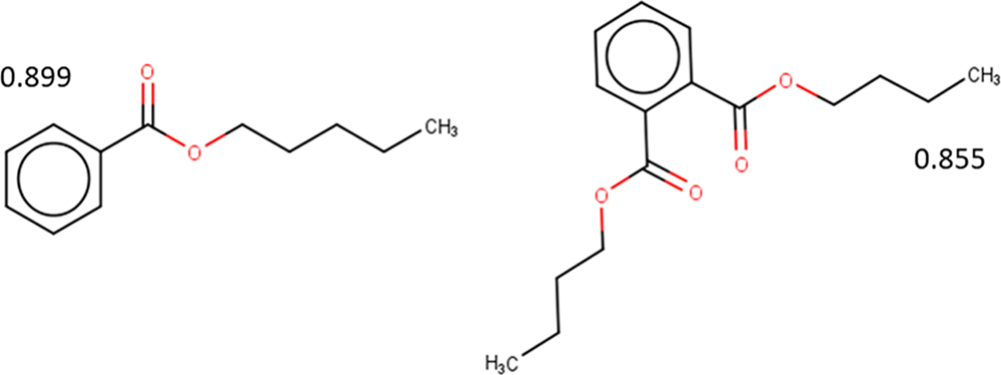
Examples of compounds identified as BPA-like based on their predicted bioactivity across 23 MIEs linked to ED.

**Figure 8:**
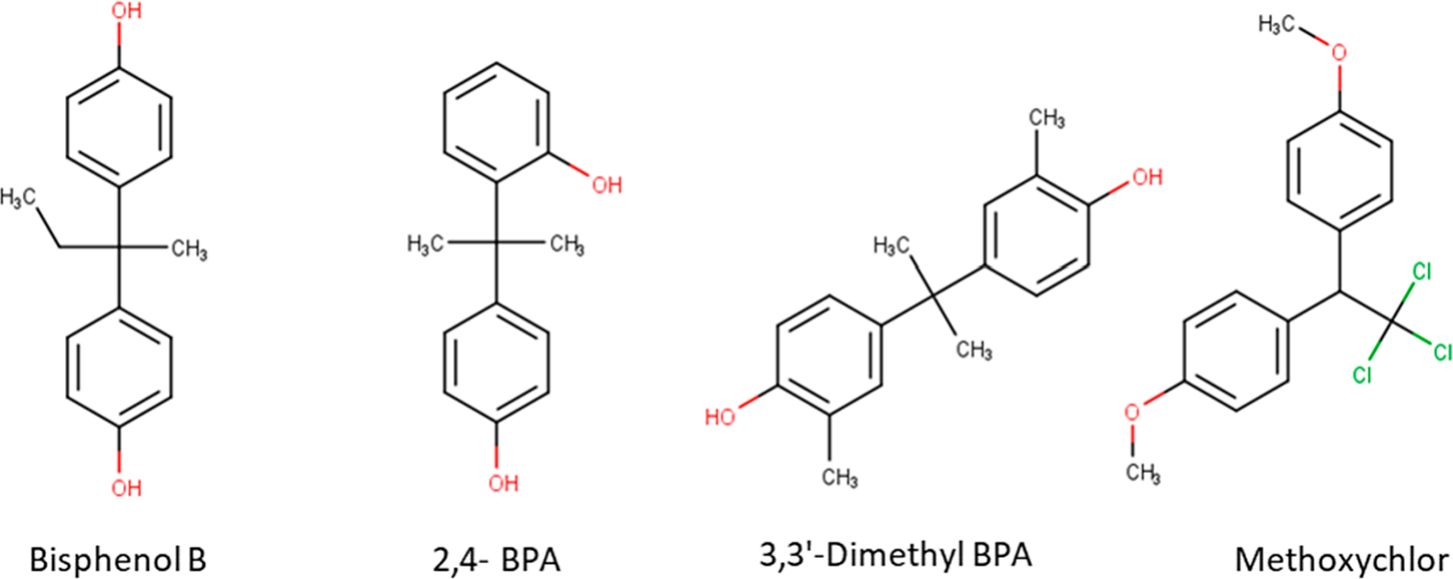
Compounds identified as DEHP-like based on their predicted bioactivity across 23 MIEs linked to ED.

The derived clusters from t-SNE and Tanimoto were compared per reference compound, and examples of overlapping compounds are presented in Figures 7 and 8 (Tables S6 and S7). More than 90% of the overlapping compounds were in the HBM4EU screening list for CECs^46^ and/or NORMAN suspect list.^47^ For BPA, comparison of the clustering outcomes highlighted 19 chemicals that were found both within the t-SNE cluster and have Tanimoto coefficients above 0.8, and some examples are presented in Figure 7. These include Methoxychlor, a compound with demonstrated endocrine activity,^48^ and Bisphenol B (BPB), a compound that has been characterized as chemical of concern due to its endocrine disrupting properties.^49^

Comparison of the DEHP clustering results highlighted four chemicals (Figure 8). All identified compounds are phthalates, which are used primarily as plasticizers. There are limited studies on the individual chemicals with respect to their mode of action; however, there is some evidence on their association with ED activity (e.g., refs (50 and 51)) and further investigations are urged.

## 4 Conclusions

With this work, *in silico* classification models have been developed for 23 MIEs that involve 14 receptors associated with endocrine-induced neurodevelopmental effects and metabolic disruption. The primary purpose of these models was to be utilized for first-tier screening, and these models address limitations due to training set imbalance and enable control over levels of confidence per prediction. Among the tested protocols for data imbalance handling, implementation of the CP framework addressed data imbalance with no compromise to model performance. The models were applied to predict activities of chemicals in a large chemical inventory (ca. 55,000 chemicals). Screening outcomes highlighted the value of quantifiable measures to assess model limitations. It was demonstrated that by following proposed strategies, it is possible to derive chemical domains with high confidence predictions of activity and highlight ‘out of domain’ compounds. Last, it was attempted to highlight chemicals of similar biological activity by combining *in silico* grouping methodologies. To do this, predicted bioactivity profiles of the chemical inventory were compared with the profiles of two well-characterized EDCs, BPA and DEPH. Grouping methodologies produced 19 and 4 compounds for their similarity with BPA and DEHP, respectively, and among the identified chemicals, several are known or suspected EDCs. The presented work could provide *in silico*-derived evidence for endocrine disrupting hazard, and the proposed strategy could provide information for prioritization and identification of suspect EDCs.

## Data Availability

Python code for CP implementation for all 23 developed models (https://zenodo.org/record/7310722#.Y5CqWnbMJXs).

## Supporting Information Available

Tables S1–S7 (XLSX)

## Abbreviations

**AhR**: aryl hydrocarbon receptor
**AR**: androgen receptor
**AUC**: area under the curve
**BPA**: Bisphenol A
**CAR**: constitutive androstane receptor
**CP**: conformal prediction
**DEHP**: bis(2-ethylhexyl)phthalate
**ED**: endocrine disruption
**EDCs**: endocrine disrupting chemical
**ER-α**: estrogen receptor alpha
**FXR**: farsenoid X receptor
**GR**: glucocorticoid receptor
**HER**: human exposure risk data set
**MCC**: Matthews correlation coefficient
**MIE**: molecular initiating events
**NPV**: negative predictive value
**PPAR-δ**: peroxisome proliferator-activated receptor delta
**PPAR-γ**: peroxisome proliferator-activated receptor gamma
**PPV**: positive predictive value
**PR**: progesterone receptor
**PXR**: pregnane X receptor
**qHTS**: quantitative high-throughput screening
**QSAR**: quantitative structure–activity relationship
**RAR**: retinoic acid receptor
**RFC**: random forest classification
**RXR**: retinoic acid receptor
**Slog*P***: log octanol–water partition coefficient
**TN**: true negative
**TP**: true positive
**TR-β**: thyroid hormone receptor beta
**t-SNE**: t-distributed stochastic neighbor embedding
**VDR3**: vitamin D3 receptor
